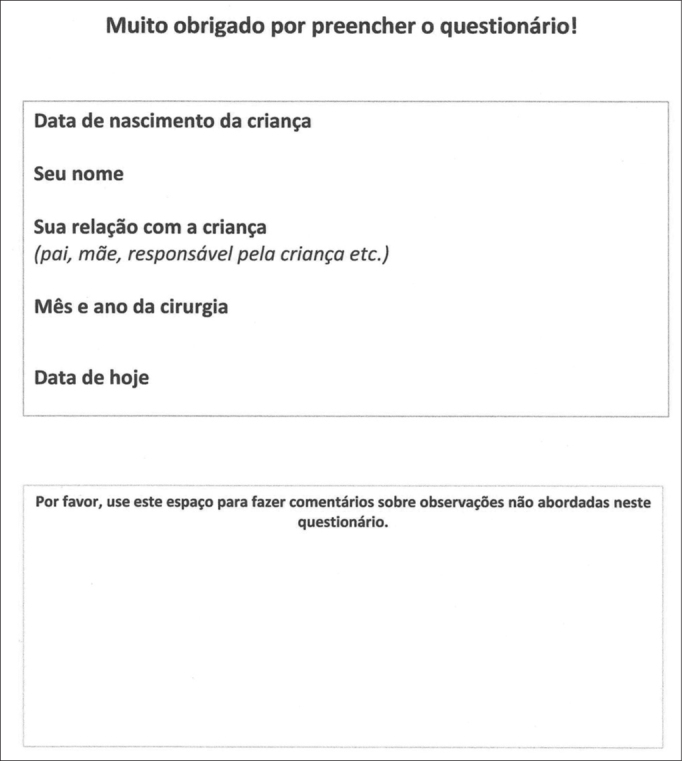# Children with cochlear implants: communication skills and quality of Life

**DOI:** 10.1590/S1808-86942012000100003

**Published:** 2015-10-20

**Authors:** Talita Fortunato-Tavares, Debora Befi-Lopes, Ricardo Ferreira Bento, Claudia Regina Furquim de Andrade

**Affiliations:** a Speech therapist, doctoral student in human communication-rehabilitation science, Medical School, Sao Paulo University.; b Speech therapist, associate professor of the Speech Therapy Course, Physical Therapy, Speech Therapy, & Occupational Therapy Department, Medical School, Sao Paulo University.; c Otorhinolaryngologist, full professor of otorhinolaryngology, Medical School, Sao Paulo University.; d Speech therapist, full professor of the Speech Therapy Course, Physical Therapy, Speech Therapy, & Occupational Therapy Department, Medical School, Sao Paulo University. Central Institute of the Clinic Hospital, Medical School, Sao Paulo University (Instituto Central do Hospital das Clínicas, Faculdade de Medicina da Universidade de São Paulo)

**Keywords:** child language, cochlear implants, hearing, quality of life, speech

## Abstract

Given the multidimensional scope of cochlear implants, there is a growing need to assess clinical measures related communicative abilities and more general aspects involved in the effectiveness of treatment, such as quality of life.

**Aim:**

To translate and adapt an international questionnaire of quality of life to Brazilian Portuguese; to apply the questionnaire in parents of children with cochlear implant to assess quality of life of children after cochlear implantation; to analyze correlations among factors related to quality of life; to analyze correlations between quality of life and clinical measures of outcome.

**Method:**

prospective study in which parents of children with cochlear implants responded to validated instruments on quality of life and communication abilities.

**Results:**

The translation and adaptation of the questionnaire was satisfactorily completed. According to the data, cochlear implants had a positive effect on quality of life of the implanted children and their families. Observed correlations for the variable communication demonstrate a direct relationship between oral communication and other variables of quality of life.

**Conclusions:**

This study makes this questionnaire available in Brazilian Portuguese. For parents of Brazilian children with cochlear implants, lexical development(acquisition and use of words) is the variable that relates most to the quality of life of their children.

## INTRODUCTION

Studies in the past 15 years have shown that the cochlear implant is an effective treatment for profound hearing loss in infants. Early restoration of auditory implant by cochlear implants significantly improve the communication skills, albeit with varied results^1^, ^2^. While most children with cochlear implants become able to attend school regularly, other remain with significantly limited verbal communication skills^3^.

It is known that the efficacy of cochlear implants depends on several factors. Many studies have searched for factors that improve the results. Duration of sensory deprivation, general development potential, possible concurrent conditions, age at surgery, anatomical/physiological and technological factors, and family involvement are examples of reported variables affecting the efficacy of cochlear implants^4^, ^5^, ^6^, ^7^.

Most studies on the impact of cochlear implants have focused on clinical assessments of efficacy (hearing and speech skills, and auditory thresholds). However, these measures are only part of the effect of cochlear implant treatment. Because of the major impact of deafness on communication, it is not clear how much clinical measures of efficacy (for instance, speech, hearing, and language measures) truly show the effectiveness of cochlear implants in general contexts (such as performance at home, at school, and in social settings). Single texts do not assess the ability of children to communicate their needs and wishes, or any improvement in self-confidence among children when interacting with normal hearing colleagues. There is evidence that clinical assessment results do not correlate with performance in unstructured settings^8^.

Because communication skills and social life often change after a cochlear implant is placed, its efficacy should be assessed taking into account structure evaluation tests and instruments for assessing the ease of daily communication, social relations, well-being, and other components of quality of life^9^, ^10^.

A need to measure results more widely has stimulated an interest in using quality of life measures for assessing the impact of cochlear implants. Thus, generic multidimensional health tools to assess the quality of life of populations at large have been widely used. These tools are not necessarily sensitive enough in audiological evaluations or to assess the results of rehabilitation, as they do not detect clinically significant improvements in users of cochlear implants^11^, ^12^. The potential psychosocial benefits of using cochlear implants – such as well-being and measures of health status – are not measured in these generic instruments^13^.

Open and closed question interviews and questionnaires or semi-structured questionnaires usually are more informative for monitoring purposes after cochlear implant surgery than generic instruments. Specific questionnaires for cochlear implant users yield information about real life situations and help describe the child's activities and participation in various social ambiences. Thus, a valid study should use adequate tools for assessing the relevant quality of life issues in the target population.

Besides being excellent research tools, questionnaires are widely used for control and quality assurance purposes in clinical settings, irrespective of interviewees – whether patients or caretakers for pediatric groups. Questionnaires help standardize information about perceptions that parents and caretakers have about implants, and inform teaching and healthcare professionals.

There are few studies in the international literature on interviews and questionnaires that investigate the expectations of parents^1^, ^2^, ^3^, ^8^, ^14^, ^15^, ^16^, their satisfaction level with implant placement^14^, ^15^, the stresses in this process^8^, and cochlear implant user and family quality of life^8^, ^14^.

The Children with Cochlear Implants: Parent´s Perspectives (CCIPP), developed by Archbold et al.^17^, is one of the most frequently used questionnaires for evaluating the quality of life in children with cochlear implants. The CCIPP is used worldwide in many cochlear implant centers (Ear Foundation, 2009) and has been described as an excellent research and clinical tool^17^, ^18^. It is a validated and reliable questionnaire that is applied when studying the experience and opinions of parents about several aspects of the quality of life of children and their families following cochlear implant surgery^19^, ^20^, ^21^.

Because of different cultures and healthcare system, changes in quality of life after cochlear implant surgery need to be studied according to cultural contexts and communication approaches. It is, therefore, extremely important to apply validated tools developed specifically for Brazilian children using cochlear implants, to assess their quality of life. Thus, the purposes of this study were:

1 – To translate and adapt the questionnaire Children with cochlear implants: parental perspectives (Ear Foundation) into Brazilian Portuguese.
2 – To apply the questionnaire to parents of children using cochlear implants and to investigate the quality of life of children and their families after cochlear implants are placed.
3 – To analyze possible correlations among factors relating to the experience and expectations of parents on the quality of life of children and the family.
4 – To analyze possible correlations among experiences and expectations of parents about the quality of life and the results of cochlear implants.

## METHODS

### Ethical issues

The institutional review board approved the study and the free informed consent form (protocol no. 342/10). All caretakers voluntarily signed the free informed consent form after agreeing to participate in the study.

### Subjects

The study sample comprised 10 infants (five male and five female) that had cochlear implants (mean age – 6 years and 2 months; standard deviation – 2.5) and their parents or caretakers. The following criteria were applied to select the sample:

1 At the time data were gathered, participants should be attending speech therapy.
2 Participants should be infants (aged from 4 to 8 years).
3 Participants should have no medical conditions or concomitant factors, such as loss of vision or compromised motor development.
4 Participants should present pre-language deafness.

Table 1 shows additional participant data.

**Table 1:** Demographic information about participants (n=10).

	Mean	Standard deviation
Age at evaluation	6.2	2.5
Age at surgery	4.6	2.2
CI use	1.6	0.9
Mean thresholds (500, 1000, 2000, and 4000Hz)	37.1	11.7
MAIS/IT-MAIS	57.6	37.4
MUSS	40.5	28.5
LDS	46.2	39

Age and CI use expressed in YY.MM. Mean thresholds expressed in dB; MAIS/IT-MAIS e MUSS expressed as %; LDS expressed in number of words

## MATERIAL

Parents or caretakers of participants answered the following instruments:

• Children with cochlear implants: parental perspectives (CCIPP)^17^.

The CCIPP questionnaire consists of 74 statements with multiple choice answers in a Likert 5-point scale: agree completely (coded 5), agree (4), neither agree nor disagree (3), disagree (2), and disagree completely (1). Forty-six statements in this questionnaire are written in a positive format and 28 are written in a negative format.

In total, 40 questions are analyzed quantitatively in subscales (general questions) consisting of three to six items. Subscale topics on child's status are: communication, general function, self-sufficiency, well-being and happiness, social relationships, and education. Subscale topics on family are: effects of the implant, and support to the child. Higher scores indicate a more positive parent perspective.

• Language Development Survey^22^ (*Lista de Avaliação Vocabulário Expressivo, or LAVE*) adapted into Brazilian Portuguese by Capovilla & Capovilla^23^.

Studies have shown that the word repertoire acquisition is delayed in children with profound hearing loss compared to normal hearing children of similar age^10^, ^18^, ^24^, ^25^. One of the reasons for this is that an anatomically and physiologically intact auditory system is a prerequisite for language acquisition and development, and its absence results in poor lexical development. Family interventions are another fundamental factor in language development; language performance in children is positively influenced by family involvement and the mother-child interactions – the linguistic input from the family, which defines the child's lexicon.

The Language Development Survey (LDS)^22^, ^26^ is frequently used to check lexical development. It comprises a checklist of words, to be filled in by parents. Capovilla & Capovilla^23^ published a translated and adapted version of the LDS in Portuguese – the name given to the test was “Lista de Avaliação de Vocabulário Expressivo or LAVE”. Although the survey is widely used, only a single study on its use in Brazilian children using cochlear implants was found^27^.

• Meaningful Use of Speech Scale (MUSS)^28^, ^29^

The Meaningful Use of Speech Scale (MUSS) questionnaire was used to analyze and measure speech skills. Nascimento^29^ translated and adapted the MUSS into Brazilian Portuguese. It consists of a structured interview of parents to assess speech use in daily situations. As with the MAIS and the IT-MAIS, the MUSS comprises 10 questions to evaluate the following areas: 1) voice control; 2) using speech without gestures or signs; and 3) using communication strategies in daily situations. Specific scoring criteria are applied to each question. The general score is obtained by adding the scores for each area.

• Meaningful Auditory Integration Scale (MAIS)^28^, ^29^ or The Infant-Toddler Meaningful Auditory Integration Scale (IT-MAIS)^29^, ^30^

Auditory skills were measured and analyzed by the Meaningful Auditory Integration Scale (MAIS) or Infant-Toddler Meaningful Auditory Integration Scale (IT-MAIS)^30^ questionnaires, according to the child's age. Both consist of structured interviews to assess the spontaneous responses of children to sounds in their daily living environments. Castiquini & Bevilaqua^31^ translated and validated the MAIS and IT-MAIS questionnaires into Brazilian Portuguese.

The assessment is based on information provided by parents to ten questions about three areas: 1) vocalization behavior; 2) alert to sounds; and 3) meaning of sounds. Specific scoring criteria are applied to each question. The general score is obtained by adding the scores for each area.

### Data analysis

Quantitative data were analyzed using the SPSS version 16 software. The adopted statistical significance was *p*<0.05. The descriptive analysis consisted of minimum and maximum measures, means, and quartiles, presented as box plots. Inferential analysis was done using a non-parametric measure of statistical dependence between two variables (Spearman's rho).

## RESULTS

### Objective 1: To translate and adapt the CCIPP^17^ questionnaire into Brazilian Portuguese

The Ear Foundation, UK, authorized the translation and adaptation into Brazilian Portuguese of the questionnaire CCIPP (in Portuguese, “*Crianças com Implante Coclear: Perspectivas dos Pais*”), according to the norms of the EORTC Quality of Life Study Group: Translation Procedure^32^.

At first, two speech therapist that were native Brazilian Portuguese speakers and fluent in English undertook two translations and cultural adaptations of the CCIPP questionnaire into Brazilian Portuguese. After comparing the two translations, both speech therapists generated a single Brazilian Portuguese version.

The final version of the questionnaire is annexed to this paper (Annex 1: CCIPP, *Crianças com Implante Coclear: Perspectiva dos Pais* – girls; Annex 2: CCIPP, *Crianças com Implante Coclear: Perspectiva dos Pais* – boys).

### Objective 2: To analyze the quality of life of children and their families after implanting cochlear implants

**Figure 1:**
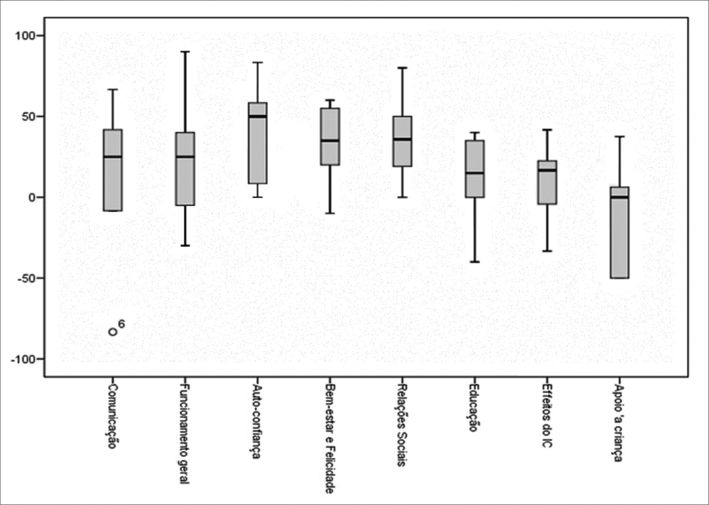
Mean values of the perception of parents in the subscales of the "Crianças com Implante Coclear: Perspectivas dos Pais" questionnaire, represented as box plots. Higher means correlate with more positive parent's perspectives.

On average, use of cochlear implants improved the quality of life of children and their families in all aspects of CCIPP subscales according to the parents (Figure 1).

Expectations of parents were more satisfactory on child self-confidence and well-being.

### Objective 3: To analyze possible correlations among CCIPP subscales

Statistically significant correlations among CCIPP subscales were found (Table 2). The largest number of correlations was found in the communication subscale – a direct relationship between communication and other quality of life variables.

**Table 2:** Correlation coefficients (Spearman's rho) among subscales of the "Crianças com Implante Coclear: Perspectivas dos Pais" questionnaire.

	Child						Family	
Child	Communication	Function in general	Self-confidence	Well-being and happiness	Social relations	Education	Effects of CI	Support to child
Communication		.903**	.888**	0. 384	0. 634*	0. 579	.830**	0. 013
Function in general			. 729*	0. 37	. 812**	0. 703*	. 892**	0. 088
Self-confidence				0. 69*	0. 391	0. 485	0. 636*	0. 194
Well-being and happiness					0. 116	0. 098	0. 194	0. 481
Social relations						0. 451	0. 667*	-0. 367
Education							. 727*	0. 203

Family								

Effects of CI								0. 126
Support to child								

* *p*<0. 05

** *p*<0. 01

### Objective 4: To analyze possible correlations among CCIPP subscales and the results of cochlear implants

Table 3 shows the correlations among CCIPP subscales and the results of placing cochlear implants. Lexical development skills (in the LDS), auditory skills (in the MAIS/IT-MAIS), and speech skills (in the MUSS) are significantly related with the communication subscale of the CCIPP from the perspective of parents.

**Table 3:** Correlation coefficients (Spearman's rho) among subscales of the "Crianças com Implante Coclear: Perspectivas dos Pais" questionnaire and the variables MAIS, LDS, MUSS, and Mean thresholds.

	Child						Family	
	Communication	Function in general	Self-confidence	Well-being and happiness	Social relations	Education	Effects of CI	Support to child
MAIS	0. 677*	0. 241	0. 603*	0. 356	0. 081	0. 421	0. 387	-0. 007
LDS	0. 583**	0. 443	0. 605*	0. 543*	0. 645**	0. 735*	0. 634**	0. 093
MUSS	0. 618*	0. 56	0. 651	0. 471	0. 644*	0. 409	0. 538	-0. 15
Mean thresholds	-0. 41	-0. 011	-0. 298	-0. 544	-0. 544	-0. 188	0. 05	-0. 071

* *p*<0. 05

** *p*<0. 01

Fewer significant correlations were seen in the development of auditory (MAIS/IT-MAIS) and speech (MUSS) skills compared to lexical development.

## DISCUSSION

The purpose of this study was to present the CCIPP questionnaire in a translated and culturally adapted version in Brazilian Portuguese. It also aimed at analyzing the quality of life of children and their families and to assess possible correlations among quality of life factors and communication skills after cochlear implants were placed.

Standardizing instruments for several countries makes it easier to compare findings across populations, which adds external validity to studies and increases knowledge on a given topic. Our results are a contribution in this direction by a translation and adaptation of the CCIPP questionnaire into Brazilian Portuguese (see Annexes). This translated questionnaire is referenced worldwide.

An analysis of Brazilian children using cochlear implants showed that parents perceive a significant effect of these implants on the quality of life of children and their families. On average, one positive effect of cochlear implants was found in all aspects of the CCIPP subscale. Parents were more satisfied with self-confidence, social relationships, well-being, happiness, general function, and communication of children. These findings corroborate those of a previous study^10^ on the five areas reported as the most satisfactory by parents after cochlear implants were placed.

In the international literature, improved self-confidence and social relationships are the earliest reported benefits of cochlear implants^33^, ^34^. Our results concur with these findings. Parental expectations in Brazil were met mostly in self-confidence, social relationships, and child well-being.

We found statistically significant correlations between several CCIPP subscales (Table 2). More correlations were found in the communication and implant effects subscales. Our findings show that parents find a directly relationship between communication and other quality of life variables – effects of cochlear implants, social relationships, self-confidence, and function in general. Additionally, these associations mean that better communication correlates with increased independence in children (better self-confidence) and improved interactions between the child and friends and family members (social relationships). The implant effect is associated with improved oral communication, more self-confidence, increased well-being and happiness, better social relationships, and a positive effect on education.

In contrast, the child support subscale did not correlate with any other subscale, meaning probably that the family supports their children irrespective of the results in other areas evaluated in this study.

From the parent's perspectives, lexical development skills (in the LDS), auditory skills (in the MAIS/IT-MAIS), and speech skills (in the MUSS) had a significant influence on the communication subscale of the CCIPP. This finding demonstrates that these instruments are mutually compatible and reinforces the validity of the Brazilian Portuguese version of the CCIPP.

Auditory thresholds were not associated with any of the quality of life subscales in this study. There were more significant correlations in lexical development skills (in the LDS) compared to development of auditory skills (in the MAIS/IT-MAIS) and speech skills (in the MUSS). Fewer relationships in auditory thresholds, and speech and auditory skill measures suggest a direct non-relation between these skills and improved quality of life, from the perspective of parents. A possible reason is that these are clinical measures and therefore not directly perceived by non-experts – the parents, for instance.

Lexical development data (in the LDS) were similar to the parent's opinions about the child's development in communication, self-confidence, well-being and happiness, social relationships, education, and effects of cochlear implants. Statistically significant correlations were found by comparing the results of LDS and the scores in each of these subscales (Table 3). These findings underline the value parents attribute to lexical development in children; our data suggest that for parents, development of a lexicon is the most closely related factor with the positive effects on the quality of life of their children.

The translated and adapted version of the CCIPP that is presented in this study was shown to be a valid instrument for use in the Brazilian pediatric population with cochlear implants. Satisfactory results were encountered, and it was compatible with other widely used instruments in this population group (such as the MAIS, IT-MAIS, and MUSS). The CCIPP provides a systematic form of investigating quality of life issues related with cochlear implant use by individuals and groups. Our proposed translation and adaptation may be applied by cochlear implant teams that intend to monitor the general effects of these devices. Individual results may yield a baseline for talks with parents, and group results can raise topics for debates among cochlear implant teams. Satisfactory results and areas of concern may be highlighted and taken into account in planning the goals of the team.

## CONCLUSION

Translation and cultural adaptation of the CCIPP questionnaire^17^ was satisfactorily concluded; this paper makes the CCIPP available in Brazilian Portuguese.

In the present study, cochlear implants had a positive effect on the lives of child users and their families. The correlations in the communication variable show a direct relationship between oral communication and quality of life.

Development of lexical, auditory, and speech activities were significantly related with the communication variable and quality of life. However, for parents of Brazilian child user of cochlear implants, quality of life aspects appear to be related more with lexical development than other communication skills that were evaluated in this study.

## Figures and Tables

**Figure:**
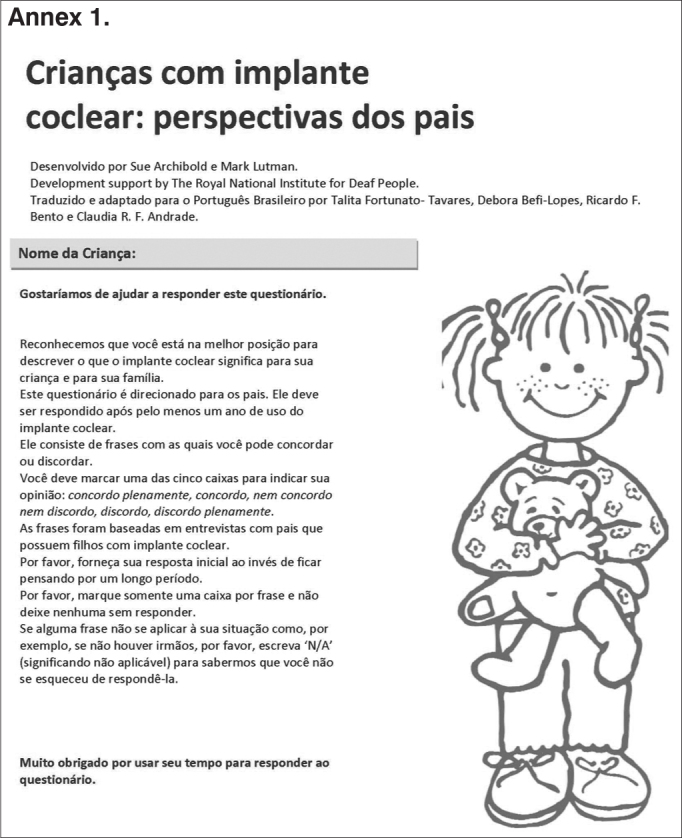


**Figure:**
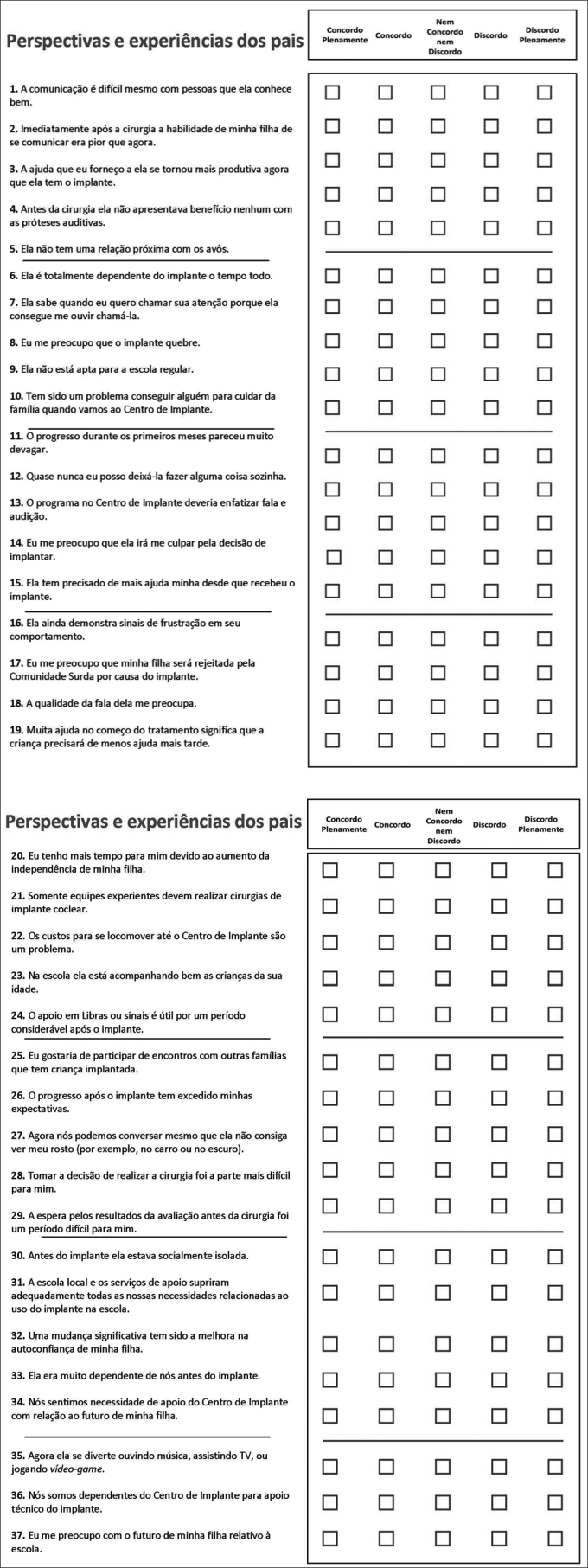


**Figure:**
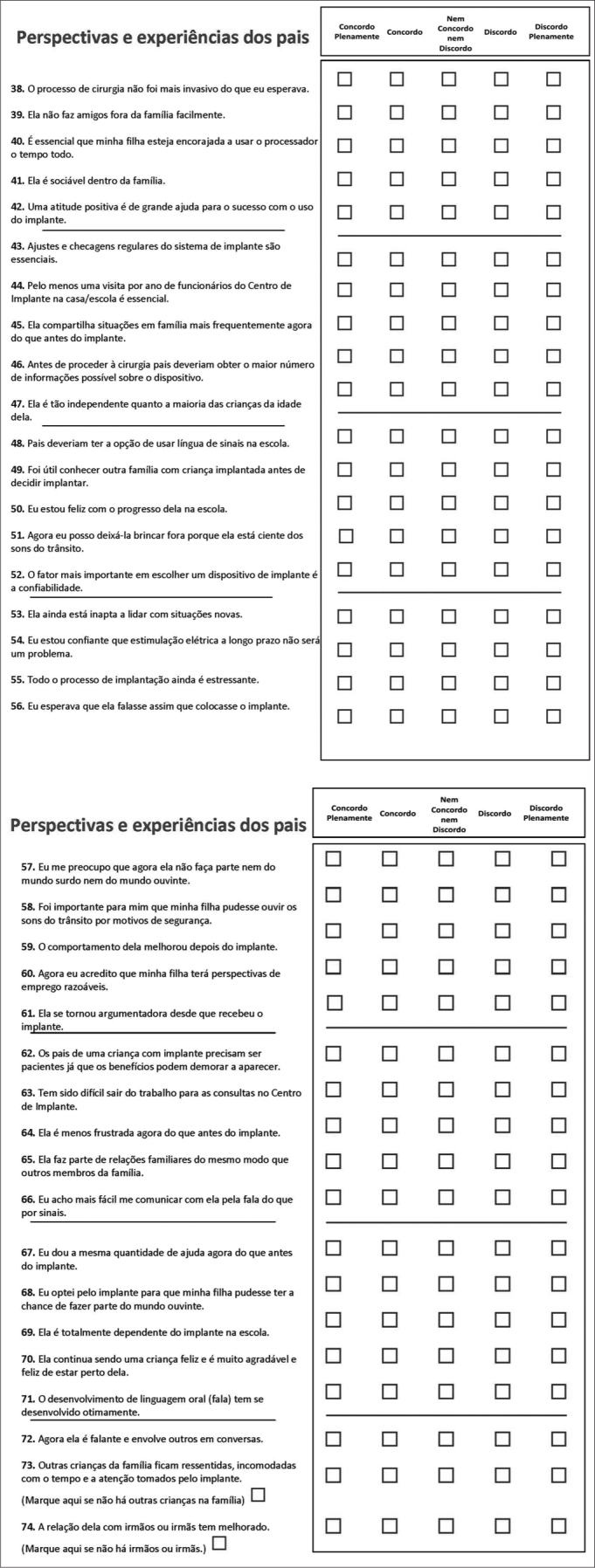


**Figure:**
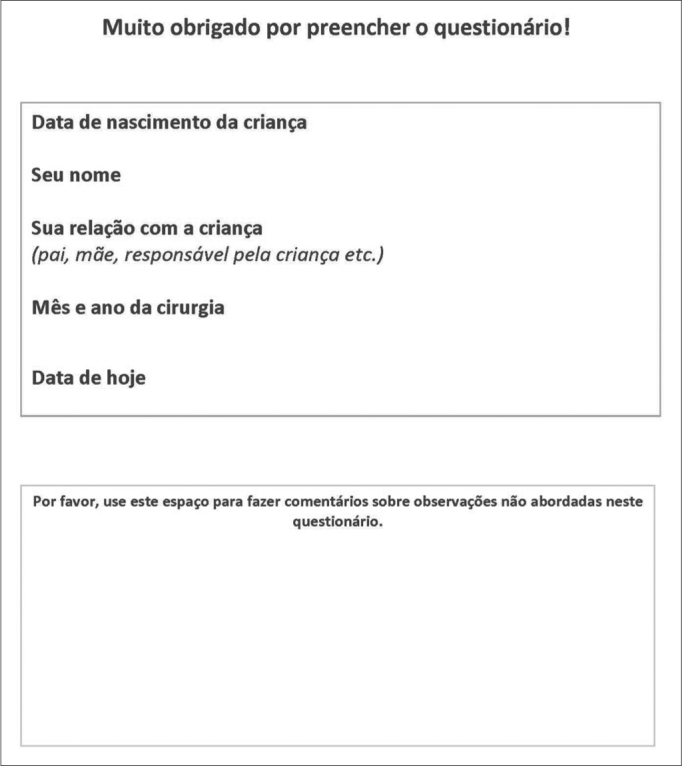


**Figure:**
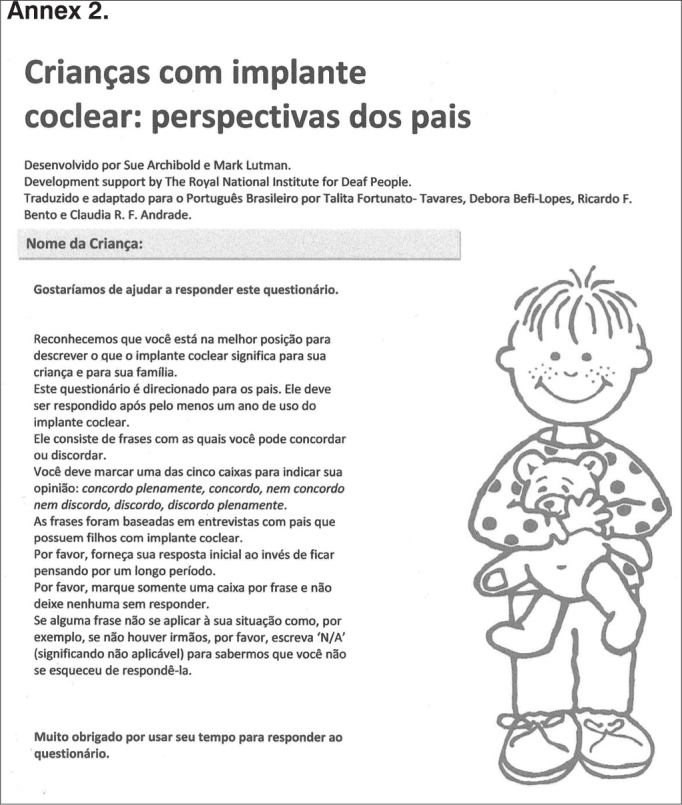


**Figure:**
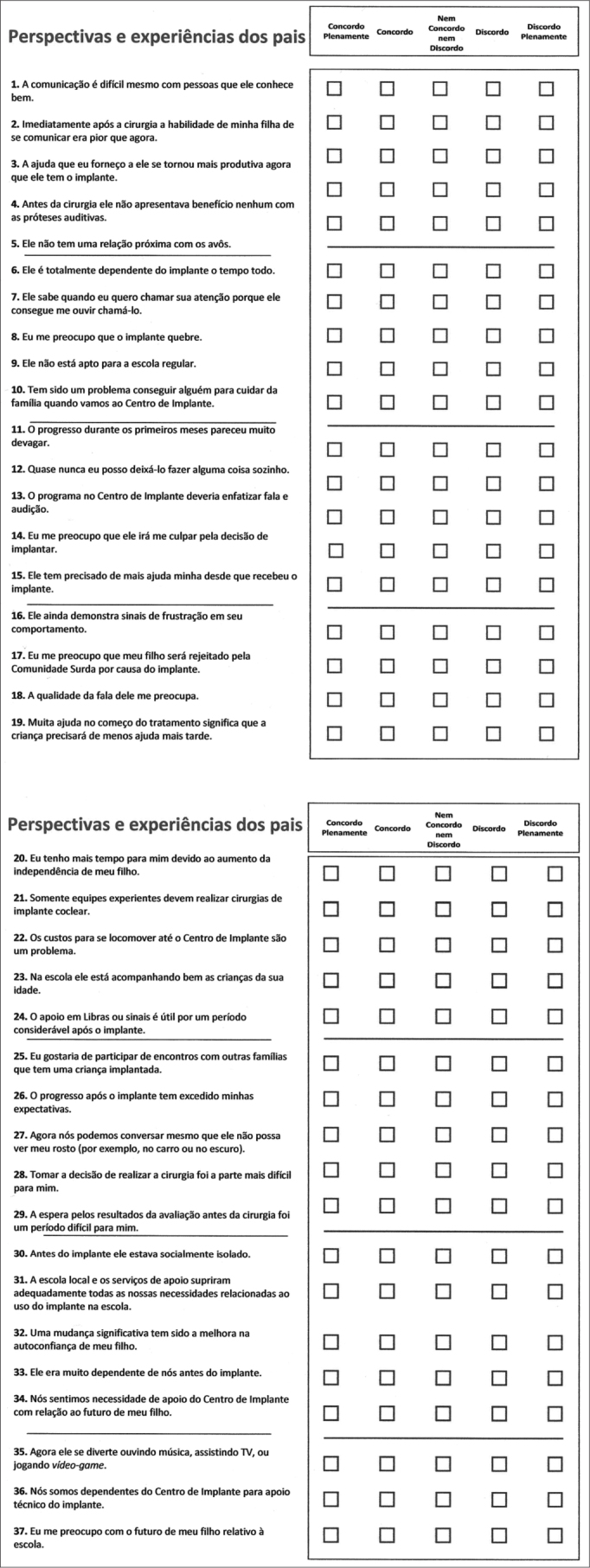


**Figure:**
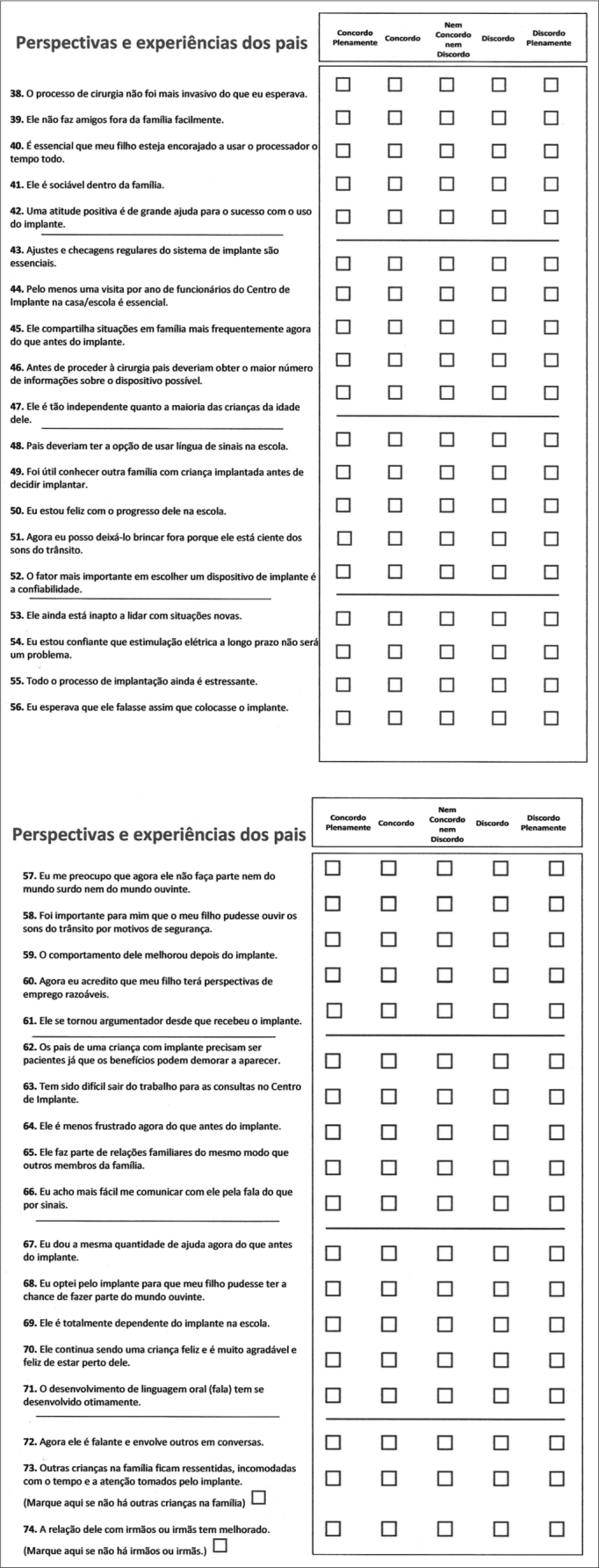


**Figure:**